# Chest CT for early detection and management of coronavirus disease (COVID-19): a report of 314 patients admitted to Emergency Department with suspected pneumonia

**DOI:** 10.1007/s11547-020-01256-1

**Published:** 2020-07-29

**Authors:** Cartocci Gaia, Colaiacomo Maria Chiara, Lanciotti Silvia, Andreoli Chiara, De Cicco Maria Luisa, Brachetti Giulia, Pugliese Silvia, Capoccia Lucia, Tortora Alessandra, Scala Annarita, Valentini Cristina, Almberger Maria, D’Aprile Maria Rosaria, Avventurieri Giacinta, Giura Riccardo, Kharrub Zaher, Leonardi Andrea, Boccia Maddalena, Carlo Catalano, Ricci Paolo

**Affiliations:** 1 Unit of Emergency Radiology, Department of Radiological, Oncological and Pathological Sciences, Umberto I University Hospital, Sapienza University of Rome, Rome, Italy; 2 Department of Psychology, Sapienza University of Rome, Rome, Italy; 3 Unit of Radiology “A”, Department of Radiological, Oncological and Pathological Sciences, Umberto I University Hospital, Sapienza University of Rome, Rome, Italy

**Keywords:** COVID-19, Chest CT, Pneumonia, Pandemic, Emergency radiology

## Abstract

**Purpose:**

The purpose of our study was to assess the potential role of chest CT in the early detection of COVID-19 pneumonia and to explore its role in patient management in an adult Italian population admitted to the Emergency Department.

**Methods:**

Three hundred and fourteen patients presented with clinically suspected COVID-19, from March 3 to 23, 2020, were evaluated with PaO2/FIO2 ratio from arterial blood gas, RT-PCR assay from nasopharyngeal swab sample and chest CT. Patients were classified as COVID-19 negative and COVID-19 positive according to RT-PCR results, considered as a reference. Images were independently evaluated by two radiologists blinded to the RT-PCR results and classified as “CT positive” or “CT negative” for COVID-19, according to CT findings.

**Results:**

According to RT-PCR results, 152 patients were COVID-19 negative (48%) and 162 were COVID-19 positive (52%). We found substantial agreement between RT-PCR results and CT findings (*p* < 0.000001), as well as an almost perfect agreement between the two readers. Mixed GGO and consolidation pattern with peripheral and bilateral distribution, multifocal or diffuse abnormalities localized in both upper lung and lower lung, in association with interlobular septal thickening, bronchial wall thickening and air bronchogram, showed higher frequency in COVID-positive patients. We also found a significant correlation between CT findings and patient’s oxygenation status expressed by PaO2/FIO2 ratio.

**Conclusion:**

Chest CT has a useful role in the early detection and in patient management of COVID-19 pneumonia in a pandemic. It helps in identifying suspected patients, cutting off the route of transmission and avoiding further spread of infection.

## Introduction

In late December 2019, a severe respiratory disease of unknown cause was reported in a cluster of patients in Wuhan City, Hubei Province, China. On January 3, 2020, a novel β-genus coronavirus, with three distinct strains, designated as 2019-nCoV, was isolated from the bronchoalveolar lavage of the affected patients and was determined to be responsible for the outbreak [1].

The outbreak was declared a Public Health Emergency of International Concern by WHO on January 30, 2020, and on February 11, 2020, the name for the new coronavirus disease was changed to COVID-19 [2]. On March 11, WHO declared the COVID-19 pandemic, and since March 10, strict quarantine rules, as in China, were imposed in Italy in order to reduce the infection peak. So far, Italy has been hit harder than any other countries in Europe with an average of 110.000 documented cases and 20.000 deaths related to severe acute respiratory syndrome (SARS) [3].

In patients with clinical features and epidemiological criteria of COVID-19, the diagnosis is established through viral nucleic acid detection in nasal or throat swabs, sputum and lower respiratory tract secretions with reverse transcription-polymerase chain reaction (RT-PCR) [4]. Although RT-PCR specificity is high, sensitivity is about 45–70%; the high rate of false negatives is probably due to low viral load or limitations of sample collection [5].

In this scenario, chest diagnostic imaging has a primary relevance in the diagnosis and severity assessment of COVID-19 together with clinical manifestations, epidemiological history and laboratory tests [5]. Chest computed tomography (CT) imaging has been demonstrated more sensitive than chest radiography (CR) to identify some of the manifestations of COVID-19 pneumonia [6, 7].

At the beginning of March, when the outbreak started in Rome, we began to combine nasopharyngeal swab specimen to chest CTs in patients with clinically suspected COVID-19 pneumonia, admitted to the Emergency Department in our regional hub hospital (Umberto I University Hospital). The purpose of our study was to assess the potential role of chest CT in the early detection of COVID-19 and to explore its role in patient management in an adult Italian population admitted to the Emergency Department with suspected pneumonia.

## Materials and methods

### Patient population

During the pandemic spread from the beginning of March 2020 in Rome, patients presented with clinically suspected COVID-19 were admitted to the Emergency Department of our regional hub hospital in a separate reserved pathway for evaluation of COVID-19 infection. Firstly, patients acceded to a pre-triage room where clinicians measured patients’ body temperature and carried out epidemiological anamnesis (travel history or contact history with individuals tested positive for novel coronavirus infection within 14 days before the onset of symptoms) and made a clinical evaluation. If the suspicious of COVID-19 persisted, patients kept on going to the separate dedicated pathway and were put in an isolation room and tested for COVID-19 with nasopharyngeal swab sample followed by RT-PCR assay to confirm the diagnosis. They also underwent blood test, arterial blood gas (ABG) examination and imaging assessment with chest CT for evaluation of COVID-19, according to our hospital’s guidelines resulting from the consent of anesthesiologists, infectivologists and radiologists as well as the Chinese guidelines available at the moment [6].

We retrospectively evaluated data from 314 patients (129 females, 185 males; mean age 59 ± 17 years) from March 3 to 23, 2020, presented with clinically suspected COVID-19. The study was approved by the Ethical Committee of Sapienza University of Rome (no. 109/2020-7/4/2020).

The mean interval between admission to the emergency triage and RT-PCR results was 10 ± 1.5 h. The time between admission to the emergency triage and CT execution was 2.5 ± 0.5 h; CT reports were produced on the spot by radiologists on duty.

Cleaning and disinfection of the CT scan room dedicated only to COVID-19 required approximately 40 min per patient.

### Imaging technique

Chest CT examinations were performed with a 64-slice scanner (Siemens SOMATOM Sensation, Siemens Medical Solutions, Forchheim, Germany) in a specific COVID-19-dedicated CT scan room of our Emergency Radiology Unit.

Patients lay in a supine position, arms raised, and were instructed to hold their breath during the acquisition, which included whole lung volume.

The acquisition parameters were set at 140 kV, 100 mAs, pitch 1.5 and collimation 0.6 mm.

To obtain high-resolution images, all data were reconstructed with a slice thickness of 1.0 mm.

### Imaging interpretation

Two radiologists (8 and 16 years of experience, respectively), who were blinded to the final diagnoses and to the RT-PCR results, evaluated chest CT scans independently. We considered nine CT findings, according to previous studies [6, 8–12]: ground-glass opacities (GGOs), consolidation, mixed GGO and consolidation, single or multiple solid nodules surrounded by ground-glass opacities (halo sign), bronchial wall thickening, air bronchogram, interlobular septal thickening, pleural effusion and mediastinal lymph node enlargement.

Ground-glass attenuation was defined as a hazy increased opacity of lung, with preservation of bronchial and vascular margins. Consolidation was defined as a homogeneous increase in pulmonary parenchymal attenuation that obscures the margins of vessels and airway walls. Bronchial wall thickening was defined in areas not close to areas of ground-glass attenuation and/or consolidation. Air bronchogram was defined as a pattern of air-filled bronchi on a background of high-attenuation airless lung. Interlobular septal thickening was defined when a septum became thicker and was clearly visible than in normal conditions. Mediastinal lymphadenopathy was judged to be present when the minimal diameter of a lymph node was larger than 10 mm [13].

The abnormalities were characterized as unilateral or bilateral. The distribution was categorized as peripheral, centrolobular, both peripheral and centrolobular, focal, multifocal and diffuse. Focal was defined as a single abnormality, multifocal as more than one abnormality and diffuse as a widespread involvement of most of the volume of one lung. Craniocaudal distribution was classified as upper lung predominant and lower lung predominant.

Clinical history of patients was available for both readers.

According to Simpson et al. [12], we classified chest CT into four categories (typical CT pattern, possible CT pattern, inconsistent CT pattern and negative for pneumonia) and subsequently into CT negative (inconsistent CT pattern and negative for pneumonia) and CT positive (typical and possible CT pattern) for COVID-19 pneumonia, as shown in Table 1. This classification helped clinicians and anesthesiologist to rapidly address patients to the intensive care unit, having all information about lung parenchyma involvement.

**Table 1:** Chest CT classification

CT findings	Imaging classification	
• Mixed GGO and consolidation pattern • Peripheral and bilateral distribution • Multifocal or diffuse abnormalities localized bilaterally • Single or multiple solid nodules surrounded by GGO (halo sign)	Typical pattern (*n* = 127)	CT positive (*n* = 171)
Absence of typical pattern AND • Single GGO opacity • Few very small GGO and consolidation pattern • Multifocal or diffuse abnormalities without peripheral distribution	Possible pattern (*n* = 44)	
Absence of typical/possible pattern AND • Isolated lobar/segmental consolidation • Smooth interlobular septal thickening with pleural effusion • Small centrolobular nodules with “ three-in-bud ” pattern	Inconsistent pattern (n = 68)	CT negative (n = 143)
No CT findings suggesting pneumonia	Negative for pneumonia (n = 75)	

## Statistical analysis

Statistical analyses were run by using SPSS (v. 25). As a first step of our analysis pipeline, we computed Cohen’s kappa for nominal variables (0 = sign was not detected; 1 = sign was detected) to assess inter-rater reliability, following the procedure by Hallgren (2012), which provides point estimates and significance tests for the null hypothesis that κ = 0 [14, 15]. Thus, using Cohen’s kappa for nominal variables (again, 0 = sign was not detected; 1 = sign was detected) we estimated the degree of agreement between reader’s radiological diagnosis and the results of RT-PCR. As a second step, we assessed the distribution of each index (i.e., CT findings) in COVID-19+ and in COVID-19− patients by computing *χ*^2^. Significance level was set after correcting multiple comparisons using Bonferroni’s correction (*p* = 0.0025). Finally, we computed point-biserial correlations between CT findings (0 = sign was not detected; 1 = sign was detected) and patient’s oxygenation status, expressed by PaO2/FIO2 ratio (obtained by ABG), in a subgroup of 94 patients. Normality of distribution in the case of PaO2/FIO2 was tested using Kolmogorov–Smirnov (KS) test.

## Results

Using RT-PCR from nasopharyngeal swab test results as a reference, we classified as COVID-19-negative (−) patients with negative RT-PCR results and as COVID-19-positive (+) patients with positive RT-PCR results. Data from 314 patients (152 COVID-19− and 162 COVID-19+) were analyzed. The two groups were matched for gender (*χ*^2^ = 0.010; p = 0.918; Cramer’s V = 0.006): 90 males and 62 females were classified as COVID-19−; 95 males and 67 females were classified as COVID-19+. Instead, COVID-19+ were older (mean age 61 ± 15 years) than COVID-19− (mean age 56 ± 18 years; Levene’s test for the equality of variance: F = 6.264, p = 0.013; *t*_298.402_ = 2.457, p = 0.015; equality was not assumed).

Time from symptoms onset to hospital admission ranged between 1 and 15 days; 115 patients presented 1–7 days after symptoms onset and the remaining 199 after the first week.

Clinical characteristics are given in Table 2.

**Table 2:** Clinical features of 314 patients

Features	No. of patients (%)
*Sex*	
Male	185 (58.9)
Female	129 (41.0)
*Age*	
Range Mean (21–40) (41–50) (51–60) (61–70) (71–80) (81–91)	20-91 59.25 48 (15.3) 49 (15.6) 51 (16.2) 63 (20.1) 56 (17.8) 47 (15.0)
*Onset symptoms*	
Fever	234 (74.5)
Cough	165 (52.5)
Dyspnea	138 (43.9)
Gastrointestinal symptoms	33 (10.5)
Astenia	16 (5.1)
Thoracic pain	12 (3.8)
Conjunctivitis	2 (0.6)
More than one symptom	212 (67.5)
None	1 (0.3)
*Underlying pathologies*	
Diabetes	21 (6.7)
Hypertension	57 (18.15)
Dyslipidemia	15 (4.8)
Cancer	7 (2.2)
Obstructive chronic bronchopulmonary disease	5 (1.6)
Heart failure	5 (1.6)
Cardiovascular and cerebrovascular disease	11 (3.5)
No underlying pathologies	213 (67.8)

Results of the inter-rater reliability are summarized in Table 3. In brief, κ was significantly higher than 0 in all indexes (all p_s_ < 0.001), suggesting that coders had a good degree of agreement.

**Table 3:** Inter-rater reliability. Intervals: 0.01–0.20 slight agreement; 0.21–0.40 fair agreement; 0.41–0.60 moderate agreement; 0.61–0.80 substantial agreement; 0.81–1.00 almost perfect or perfect agreement

Index	Kappa	*t*	*p*	Intervals
Ground-glass opacity (GGO)	0.508	9.011	0.000000	Moderate agreement
Consolidation	0.410	7.266	0.000000	Moderate agreement
Mixed GGO and Consolidation	0.664	11.799	0.000000	Substantial agreement
Single/multiple nodules with halo sign	0.519	9.204	0.000000	Moderate agreement
Peripheral distribution	0.212	4.823	0.000001	Fair agreement
Centrolobular distribution	0.190	3.492	0.000480	Slight agreement
Both peripheral and centrolobular distribution	0.239	5.920	0.000000	Fair agreement
Upper lung	0.306	5.578	0.000000	Fair agreement
Lower lung	0.566	10.057	0.000000	Moderate agreement
Both upper lung and lower lung	0.733	13.034	0.000000	Substantial agreement
Unilateral	0.643	11.388	0.000000	Substantial agreement
Bilateral	0.853	15.113	0.000000	Almost perfect agreement
Focal	0.530	9.415	0.000000	Moderate agreement
Multifocal	0.676	12.060	0.000000	Substantial agreement
Diffuse	0.500	9.215	0.000000	Moderate agreement
Interlobular septal thickening	0.416	7.412	0.000000	Moderate agreement
Bronchial wall thickening	0.269	5.022	0.000001	Fair agreement
Air bronchogram	0.500	9.661	0.000000	Moderate agreement
Lymph nodes	0.404	8.107	0.000000	Fair agreement
Pleural effusion	0.709	12.573	0.000000	Substantial agreement
COVID Positive according to CT findings	0.814	14.419	0.000000	Almost perfect agreement

**Fig. 1:**
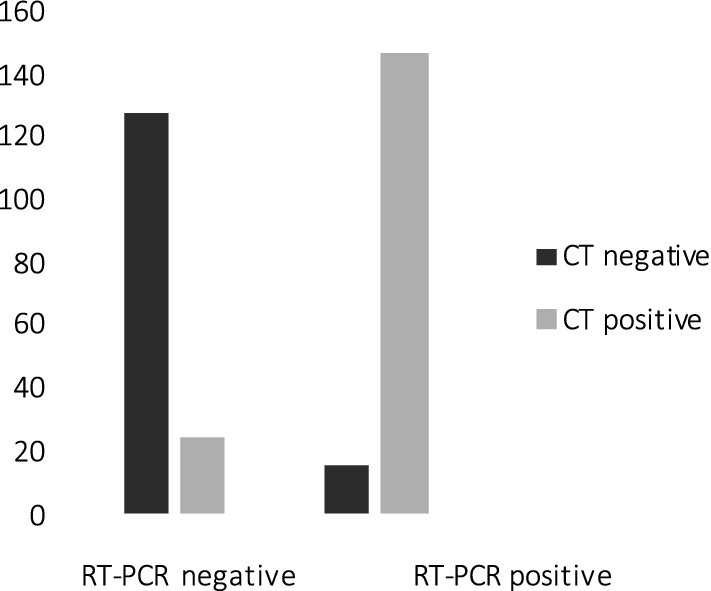
Agreement and discrepancies between CT findings and RT-PCR: 147 COVID-19+ were CT positive; 128 COVID-19− were CT negative; 24 cases were CT positive with negative RT-PCR results; 15 cases were CT negative with positive RT-PCR results

Thus, the following analyses were run on one of the two raters. First, we estimated the degree of agreement between reader’s radiological diagnosis and the results of RT-PCR, finding substantial agreement between the two measurements (*κ* = 0.751, *t* = 13.328, *p* < 0.000001) with a total of 147 COVID-19+ with CT positive and 128 COVID-19− with CT negative. We also found discrepancies in 24 cases having CT positive, but negative RT-PCR results and in 15 cases with CT negative, but positive RT-PCR results, as shown in Fig. 1.

**Fig. 2:**
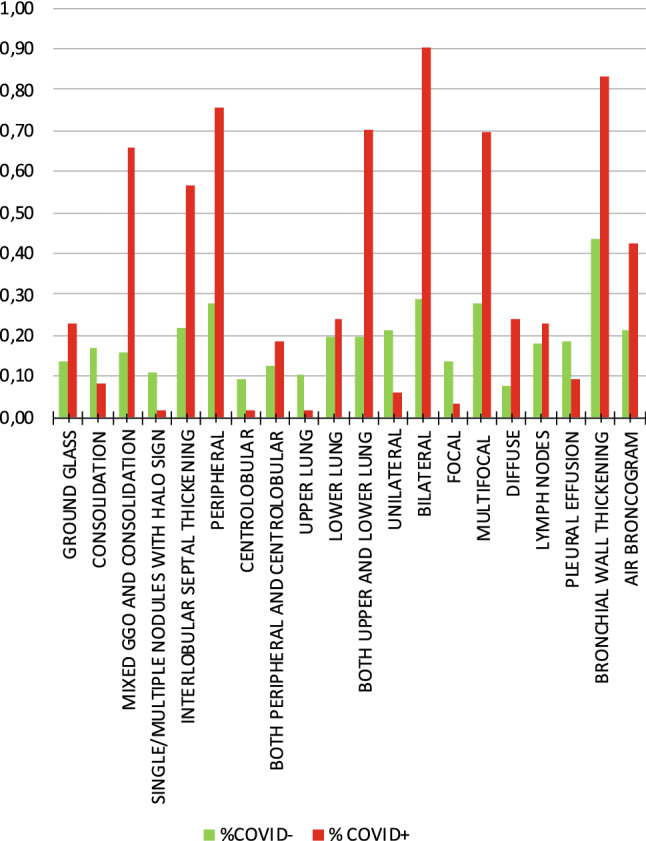
Percentage of COVID-19+ and COVID-19− for each radiological index

**Fig. 3:**
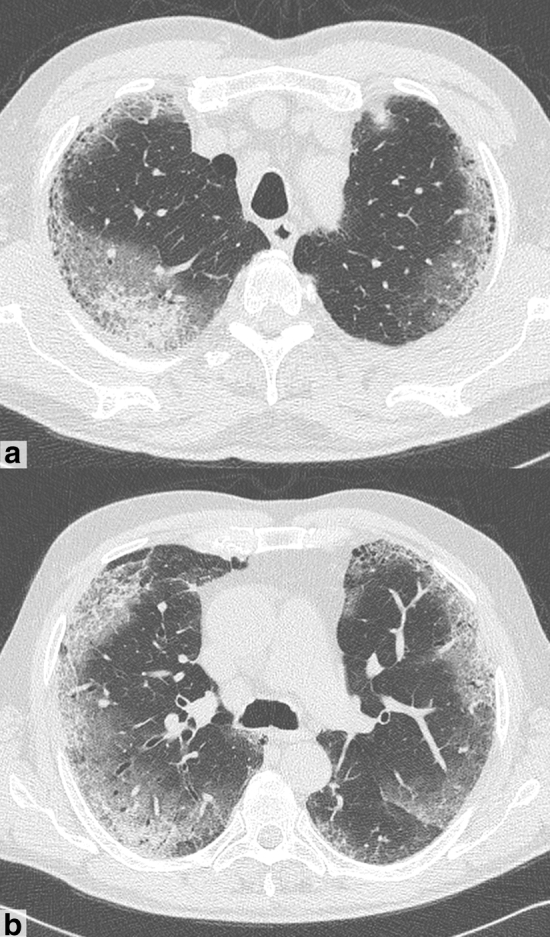
A 75-year-old man presented with fever and dyspnea in the last 13 days, COVID+. **a–b** CT shows diffuse bilateral ground-glass opacities with prevalent peripheral distribution, septal thickening and small areas of consolidation with air bronchogram (mixed GGO and consolidation pattern)

The distribution of each radiological index (i.e., CT findings) in COVID-19+ and in COVID-19− is reported in Table 4 (Fig. 2). In brief, mixed GGO and consolidation pattern, with peripheral and bilateral distribution, multifocal or diffuse abnormalities localized in both upper lung and lower lung, in association with interlobular septal thickening, bronchial wall thickening and air bronchogram, showed higher frequency in COVID-19+. Data are also reported in Fig. 3 as the percentage of COVID-19+ and COVID-19−, showing each radiological index.

**Table 4:** Distribution of radiological indexes (i.e., CT findings) in COVID+ and COVID−

Index	χ^2^	p	Cramer’s V	Direction
Ground glass	4.240	0.039476	0.116	NA
Consolidation	5.945	0.014763	0.138	NA
Mixed GGO and Consolidation	81.472	0.000000*	0.509	Positive
Single/multiple nodules with halo sign	11.452	0.000714*	0.191	Negative
Peripheral distribution	71.445	0.000000*	0.477	Positive
Centrolobular distribution	8.292	0.003981	0.163	NA
Both peripheral and centrolobular distribution	2.157	0.141933	0.083	NA
Upper lung	10.380	0.001274*	0.182	Negative
Lower lung	0.860	0.353628	0.052	NA
Both upper lung and lower lung	80.975	0.000000*	0.508	Positive
Unilateral	14.985	0.000108*	0.218	Negative
Bilateral	122.822	0.000000*	0.625	Positive
Focal	11.887	0.000565*	0.195	Negative
Multifocal	55.663	0.000000*	0.421	Positive
Diffuse	15.090	0.000103*	0.219	Positive
Interlobular septal thickening	40.274	0.000000*	0.358	Positive
Lymph nodes	1.245	0.264454	0.063	NA
Pleural effusion	5.569	0.018277	0.133	NA
Bronchial wall thickening	54.228	0.000000*	0.416	Positive
Air bronchogram	16.675	0.000044*	0.230	Positive

Significant differences are marked with an asterisk: Significance level was set after computing Bonferroni’s correction for multiple comparisons (*p* < 0.0025). The direction of the effect summarizes which group shows higher probability of distribution for each radiological index (positive = higher frequency in COVID+; negative = higher frequency in COVID−)

Finally, we found a significant correlation between PaO2/FIO2 values (which were normally distributed; KS = 0.042; *p* = 0.200) and the following CT indexes: mixed GGO and consolidation pattern (*r* = − 0.431; *p* = 0.000), bilateral (*r* = − 0.212; *p* = 0.020), diffuse (*r* = − 0.473; *p* = 0.000), both peripheral and centrolobular distribution (*r* = − 0.412; *p* = 0.000) and both upper lung and lower lung (*r* = − 0.337; *p* = 0.000). Also, interlobular septal thickening (*r* = − 0.435; *p* = 0.000), bronchial wall thickening (*r* = − 0.431; *p* = 0.000) and air bronchogram (*r* = − 0.383; *p* = 0.000) showed a significant correlation with oxygenation impairment. Correlation between CT positive and PaO2/FIO2 ratio was statistically significant (*r* = − 0.241; *p* = 0.010) as well.

## Discussion

The pandemic spread of coronavirus from China to Italy has represented a major problem due to overwhelming transmission and severity of disease, which is a potential threat to the healthcare system due to the limited availability of hospital resources, especially with regard to intensive care units [16]. The purpose of this study was to assess the potential role of chest CT in the early detection of COVID-19 and to explore its role in patient management in an adult Italian population admitted to the Emergency Department with suspected pneumonia.

We comprehensively evaluated and analyzed the CT findings of 314 patients admitted to the Emergency Department of our regional hub hospital in Rome, Italy. Using RT-PCR from nasopharyngeal swab test results as a reference, as mentioned in the results section, we classified as COVID-19− patients with negative RT-PCR results and as COVID-19+ patients with positive RT-PCR results.

Nasopharyngeal swab test is a widely used method to confirm COVID-19 infection, and it is recommended by WHO guidelines, which state that a clinically suspected case is confirmed only in the presence of a positive RT-PCR result [5]. Despite specificity, swab tests only have limited sensitivity and negative predictive value and are not suitable to assess disease severity [4, 17, 18]. Moreover, results of swab tests are available with a lag of several hours. In our study, mean turnaround time for swab results was 10 h, while suspected patients must remain in isolation, hospitalized and mostly under clinical surveillance.

Even if chest radiography (CXR), performed using portable imaging equipment, has been considered the first-line examination, due to the easy equipment disinfection, the bedridden patients accessibility [19] and the capability to differentiate between a normal and severely abnormal chest, chest X-ray findings have a lower sensitivity than initial RT-PCR testing compared to CT (69% versus 91%, respectively), particularly at an early stage of the disease [20]. Chest CT, due to the reported high sensitivity and specificity [21], is an accurate imaging modality in symptomatic patients at admission, to assess disease severity and guide patient management [22], and it is considered a reliable imaging modality for diagnosis and for monitoring the care of patients with COVID-19 pneumonia, especially in an emergency setting where timing is crucial for early identification of diseased patients and the separation of disease-free patients from suspected disease, in order to reduce human-to-human transmission [9]. Our results showed a substantial agreement between RT-PCR results and CT findings (*p* < 0.000001), as well as an almost perfect agreement between the two readers.

In patients with COVID-19+ (*n* = 162), we observed a total of 147 cases classified as CT positive, according to findings interpretation. In this group, mixed GGO and consolidation pattern with peripheral and bilateral distribution, multifocal or diffuse abnormalities localized in both upper lung and lower lung, in association with interlobular septal thickening, bronchial wall thickening and air bronchogram, showed higher frequency (Fig. 3). Unlike previous studies that show prevalence of GGO pattern at the early CT scan, the mixed GGO and consolidation were the most common patterns in our study [23]. This may be due to the fact that in Italy, paucisymptomatic patients have mostly been managed at home by general practitioners, and only if patients worsen, they are sent to the Emergency Department. Most of our COVID+ population reached the hospital 7-15 days after onset of symptoms. For the same reason, in our study the most common distribution is both peripheral and centrolobular because during the second week the disease can spread and involve even the central regions [24]. CT features of COVID-19 pneumonia are similar to other common viral pneumonia [7]. However, according to other studies [25], we observed that spatial distribution, as well as attenuation pattern, could be suggestive of COVID-19 pneumonia [24, 26]. Besides, CT imaging interpretation of symptomatic patients during the days of the peak of the pandemic spread in Rome (March 3–23, 2020) could have affected our final diagnosis by including in the CT-positive group also the consistent, but less typical patterns (possible CT pattern—Table 1). This diagnostic approach is probably not to be extended in settings different from pandemic outbreak where other causes of interstitial pneumonia must be taken into account [27, 28].

Despite substantial agreement, we also had some discrepancies between CT and RT-PCR.

**Fig. 4:**
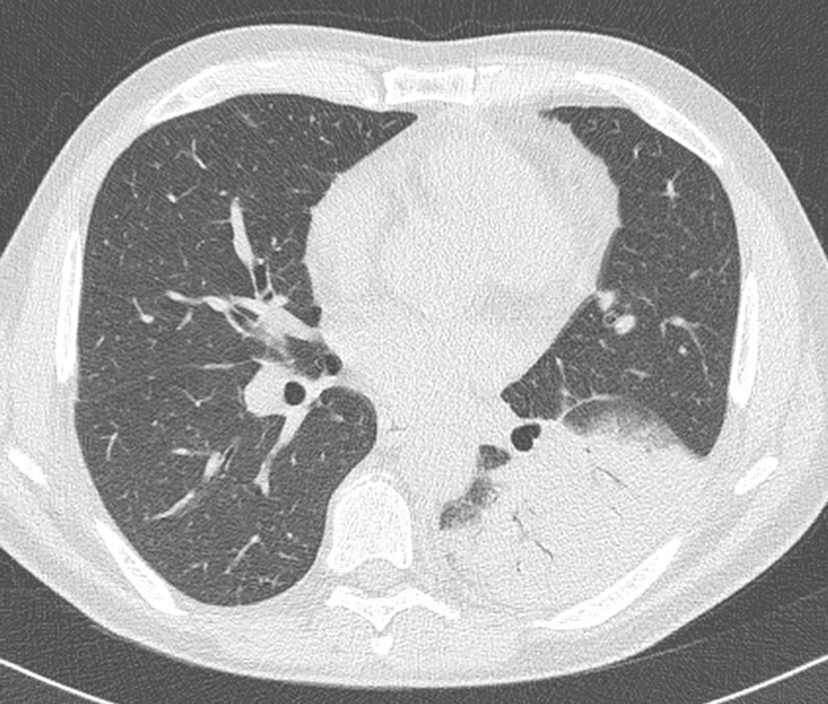
A 21-year-old man with dyspnea, cough and thoracic pain in the last two days, without fever and without history of COVID-19 exposure. CT shows a large area of consolidation with air bronchogram involving the lower lobe of the left lung suggesting bacterial lobar pneumonia. The patient, instead, was positive to the RT-PCR test

In 15 cases, RT-PCR showed positive results in spite of CT negative. In this subgroup: One patient showed a lobar uniform consolidation strongly suggestive of lobar pneumonia (i.e., as seen in streptococcus pneumonia) and was interpreted as bacterial pneumonia (Fig. 4); one patient had neoplastic history and his lung alterations were interpreted as metastatic involvement with lymphangitic carcinomatosis; and four patients showed only a single and subtle opacity that was considered an atypical CT finding, not suggestive of COVID-19 pneumonia. In the remaining nine patients, both radiologists found no parenchymal abnormalities (normal chest CT). Anyhow, in these nine patients, chest CT ruled out the presence of pulmonary involvement in an emergency context and allows clinicians to treat them conservatively. Most were discharged under strict “active surveillance.” Thus, chest CT even when negative had importantly affected clinical management in suspected infected patients. Hence, a normal CT scan in the presence of a positive swab test could be a good prognostic indicator of the absence of pulmonary involvement. A secondary hypothesis to explain why a normal CT scan could be found associated with a positive swab is that the CT scan has been performed too early, before the development of pulmonary involvement, because frequency of CT findings is dependent on infection time course [29]. In fact, early reports have stated that initial imaging might show normal findings in 15% of individuals, so a normal chest imaging examination does not exclude the infection [7]. Moreover, in a study conducted in China during the first 2 months of outbreak, no CR or CT abnormality was found in 17.9% patients with non-severe disease and in 2.9% patients with severe disease [30]. A better understanding of the spectrum of the disease is needed, since the same study revealed that in 8.9% of the patients, 19-nCoV infection was detected before the development of viral pneumonia or viral pneumonia did not develop.

**Fig. 5:**
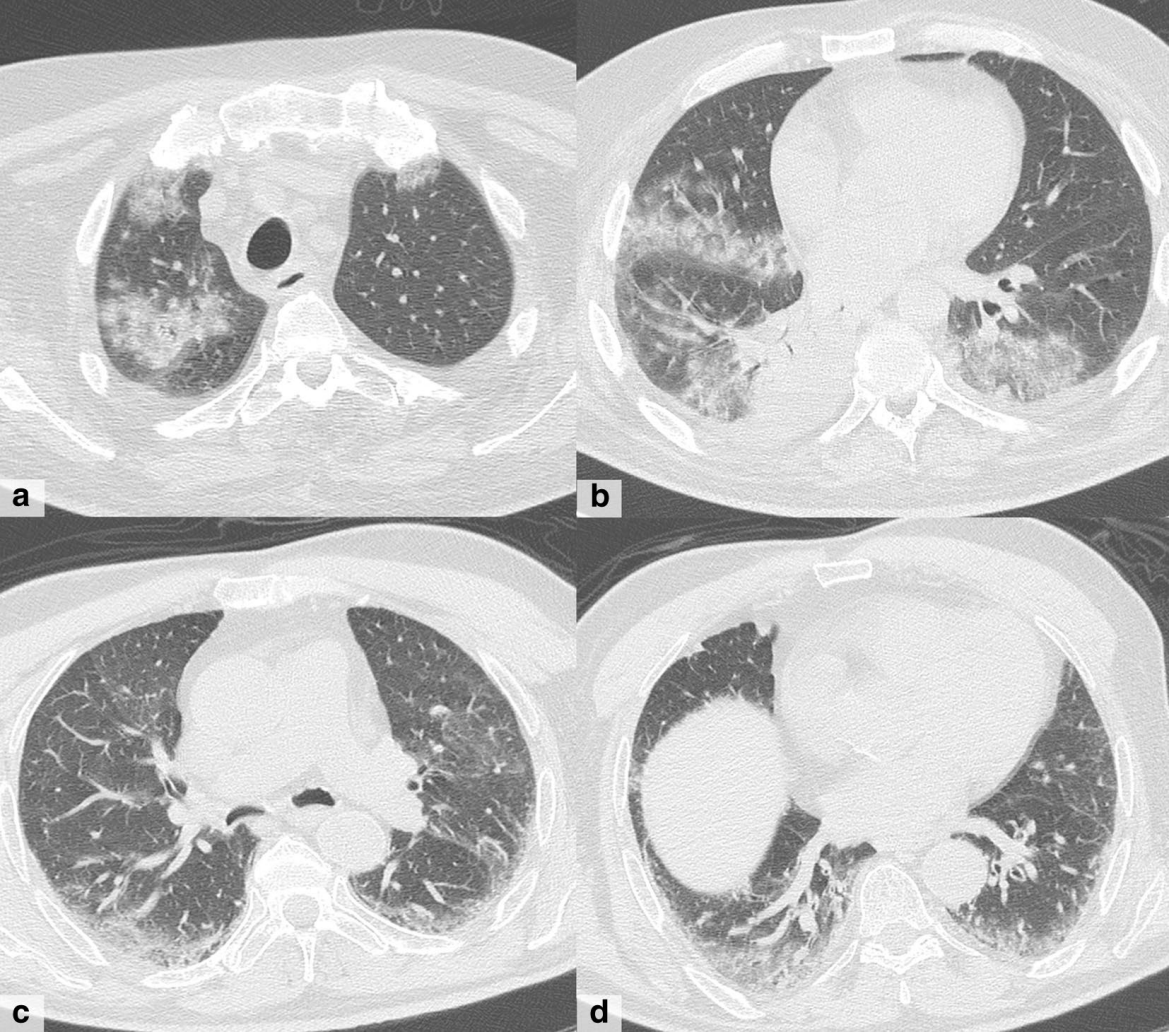
Two cases of discrepancies between CT findings and RT-PCR results. **a–b** A 57-year-old man with diabetes and hypertension, admitted in critical condition for serious dyspnea and stupor. CT shows bilateral and mostly peripheral multifocal confluent areas of ground-glass opacity with a wide area of consolidation in the lower right lung **(b)**. RT-PCR result was negative, and the final diagnosis was Klebsiella pneumoniae infection. **c–d** A 83-year-old man with cardiomyopathy and diabetes who presented with fever in the last 3 days and history of COVID-19 exposure. CT shows thin semilunar symmetric areas of peripheral subpleural increased density, bronchial wall thickness, signs of vascular congestion and cardiomegaly; these signs were interpreted as congested interstitial spaces and poorly aerated zones secondary to bronchitis and heart dysfunction. Instead, RT-PCR result was positive

In 24 cases with negative swab tests (COVID -), CT findings were consistent with COVID-19 pneumonia (CT positive). In this subgroup, one patient had pneumonia from another cause (Klebsiella pneumoniae) and two patients had chronic bronchitis with disventilatory alterations of lung parenchyma, mimicking bilateral ground-glass opacities (Fig. 5). Lung infections or inflammatory conditions can share some findings with COVID-19 pneumonia, and correct interpretation of those has probably been mistaken in a context of pandemic spread where most patients with fever and respiratory symptoms are expected to be affected by COVID-19.

**Fig. 6:**
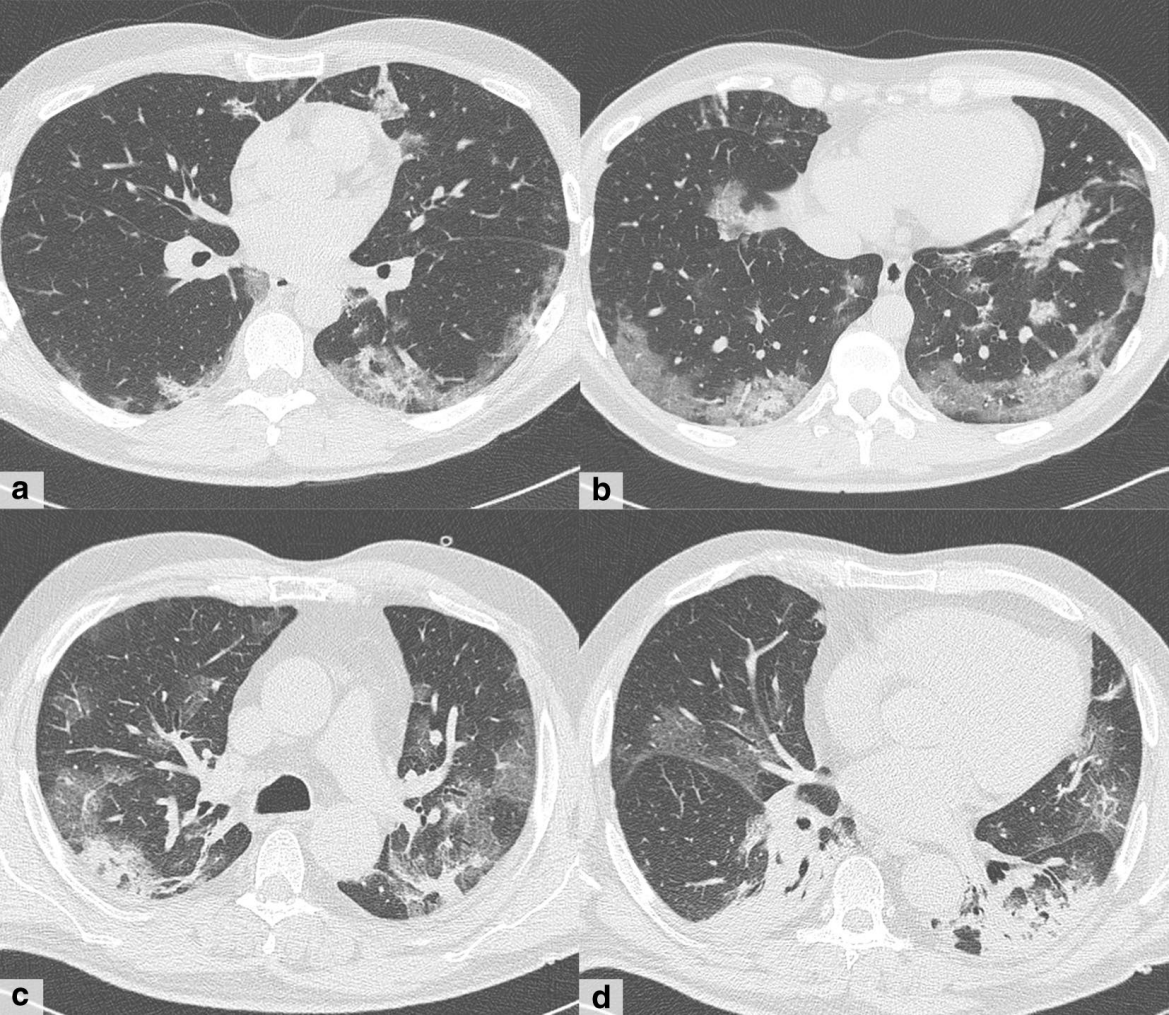
**a**–**b** CT scan in a 43-year-old man with fever and cough in the last 12 days shows the most typical and frequent features of COVID-19 pneumonia: bilateral multifocal and confluent ground-glass opacities in a peripheral subpleural distribution, associated with consolidation area in the left lower lobe. The patient was positive to RT-PCR test. **c–d** A 64-year-old man with cough and dyspnea for 10 days, treated at home with antibiotics without benefit and arrived to the hospital for the onset of fever in the last day. CT scan shows similar pattern and distribution of patient in figures a, b, but the first two swabs were negative. Anyhow, he was hospitalized and treated as a positive patient. The RT-PCR test turned positive only on the third sample

In the remaining 21 cases, we observed highly suggestive CT findings for COVID-19 pneumonia, though negative RT-PCR results. This was not confirmed in all patients because six patients had positive swab test results in following repeated samples; thus, in this subgroup of patients CT scan actually allowed an early diagnosis (Fig. 6). Since repeated swab tests are performed after 24 h from first one, and if the latter is still negative, a third is performed the following day and so on, CT scans can give remarkable diagnostic anticipation. This is in accordance with previous experiences, and it is already known that some patients with positive chest CT findings may at first present negative swab test; thus, repeated sampling may be required in patients with high clinical suspicion and positive CT findings [31]. Our experience confirms that when swab tests are negative, the possibility of a false-negative result should be considered in the context of a patient’s recent exposures and the presence of clinical and radiological signs and symptoms consistent with 2019-nCoV infection. For this reason, in case of epidemiological anamnesis and CT findings suggestive of COVID-19, repeated swab test and patient isolation should be considered [32, 33]. Reasons for false-negative RT-PCR may include insufficient cellular material for detection and improper extraction of nucleic acid from clinical materials [34].

Finally, as demonstrated in the correlation analysis, we found that chest CT considered “CT positive” and specific CT findings mentioned in the result section, significantly correlated with oxygenation impairment, expressed by PaO2/FIO2 ratio. While most people with COVID-19 develop only mild or uncomplicated illness, approximately 14% develop severe disease that require hospitalization and oxygen support and 5% require admission to an intensive care unit [35]. In latter cases, COVID-19 can be complicated by the acute respiratory distress syndrome (ARDS*)* [36]. A draft definition accepted worldwide proposed three mutually exclusive categories of ARDS based on degree of hypoxemia: mild (PaO2/FIO2 ≤ 300 mm Hg), moderate (PaO2/FIO2 ≤ 200 mm Hg) and severe (PaO2/FIO2 ≤ 100 mm Hg) [37]. As oxygenation impairment increases, several therapeutic options must be considered, like high-flow nasal oxygen in mild–moderate ARDS or endotracheal intubation and mechanical ventilation in severe cases [38].

Our study confirmed a strong correlation between swab test and chest CT findings for diagnosing or ruling out COVID-19 pneumonia and a strong relationship between clinical variables like hypoxemia and CT findings in patients considered CT positive. Hence, our results suggest the creation of a flowchart for managing patients admitted to the Emergency Department with suspected infection from 2019-nCoV.

Patients with negative CT scan can be early discharged and isolated at home considering the low likelihood of a positive swab test and the very unlikely development of pulmonary problems. CT may help in screening out patients with suspected disease, especially patients with an initial negative RT-PCR screening result [31]. However, it must always be kept in mind that low sensitivity and negative predicted value of chest CT in early patients limit its role as an effective standalone tool to rule out COVID-19 [29]. Conversely, patients with positive CT scan reached an early diagnosis compared to the results of the RT-PCR results which can lag at least 8 h. Although CT imaging has a certain turnaround time, nucleic acid and gene sequencing detection require a relatively longer time compared to CT. Therefore, chest CT represents a valuable tool in identifying patients with 2019-nCoV infections at an early stage, when clinical symptoms may be unspecific or sparse [25]. Thus, for the timely and accurate diagnosis of COVID-19, CT can quickly identify suspected patients and significantly help in isolating the source of infection, cutting off the route of transmission and avoiding further spread [34].

The above-described management improves clinical decision making, especially in the emergency setting where it is of paramount importance to stratify outpatients in suspected or non-suspected cases, while waiting for the RT-PCR results [39]. A management strategy based on CT results and clinical condition has already been used during the COVID-19 epidemic in China, when 10567 patients were treated as clinically diagnosed cases. This designation has been used in Hubei Province. In these cases, no RT-PCR test was performed but diagnosis was made based on typical symptoms, exposure history and chest CT manifestations consistent with COVID-19 pneumonia. Under these criteria, 10,567 cases were diagnosed and isolated. This strategy quarantined a large number of suspected people and protected the healthy people to a major extent [16].

Our study has limitations. We have not made a comparison with X-rays, which have been rarely performed according to our hospital’s guidelines because the Unit of Emergency Radiology has a dedicated CT room for suspected COVID-19 patients; as a result, this diagnostic strategy probably cannot be adopted in all spoke hospitals in the region. In addition, according to SIRM (Italian Society of Medical and Interventional Radiology) chest CT scan is recommended in symptomatic patients [40].

An intrinsic limit of a cohort of patients admitted to the Emergency Department is a large inhomogeneity, since the time of disease onset was unknown, depending on different incubation times. Moreover, disease severity was different among patients depending on previous clinical conditions that can affect lung pathology. According to the limited duration of this study (3 weeks), we still do not have long-term follow-up data which could clarify whether the small amount of normal chest CTs in the presence of positive swab tests were indicative of a preclinical lung disease or were to be considered as false-positive swab tests.

In conclusion, our study shows how chest CT has a useful role in the early detection of COVID-19 pneumonia in a pandemic. Chest CT is particularly helpful in patient management in an Emergency Department because it can reliably identify suspected patients and significantly help in isolating the infected ones, cutting off the route of transmission and avoiding further spread of infection.

## Abbreviations

2019-nCoV: 2019 new coronavirus
COVID-19: Coronavirus disease 2019
RT-PCR: Reverse transcription-polymerase chain reaction
CT: Computed tomography
GGO: Ground-glass opacity
